# Cell-Cycle-related Protein Centromere Protein F Deficiency Inhibits Cervical Cancer Cell Growth by Inducing Ferroptosis Via Nrf2 Inactivation

**DOI:** 10.1007/s12013-024-01251-7

**Published:** 2024-03-27

**Authors:** Xin hui Tang, Tian nan Zhao, Li Guo, Xin yue Liu, Wei na Zhang, Ping Zhang

**Affiliations:** 1 Department of Gynecology, Qingdao Municipal Hospital, Qingdao, 266011 China; 2 Department of Obstetrics and Gynecology, Putuo People’s Hospital, School of Medicine, Tongji University, Shanghai, 200060 China; 3 Dalian Medical University, School of Graduate, Dalian, 116000 China; 4 Department of Gynecology, Changzhi People’s Hospital, Changzhi, 046000 China

**Keywords:** Cervical cancer, CENPF, Cell cycle, Ferroptosis, Nuclear factor E2-related factor 2

## Abstract

Cervical cancer (CC) is one of the severe cancers that pose a threat to women’s health and result in death. CENPF, the centromere protein F, plays a crucial role in mitosis by regulating numerous cellular processes, such as chromosome segregation during mitosis. According to bioinformatics research, CENPF serves as a master regulator that is upregulated and activated in cervical cancer. Nevertheless, the precise biological mechanism that CENPF operates in CC remains unclear. The aim of this study was to analyze the function of CENPF on cervical cancer and its mechanism. We conducted immunohistochemistry and western blot analysis to examine the expression levels of CENPF in both cervical cancer tissues and cells. To explore the hidden biological function of CENPF in cell lines derived from CC, we applied lentivirus transfection to reduce CENPF manifestation. CENPF’s main role is to regulate ferroptosis which was assessed by analyzing Reactive Oxygen Species (ROS), malonaldehyde (MDA), etc. The vitro findings were further validated through a subcutaneous tumorigenic nude mouse model. Our research finding indicates that there is an apparent upregulation of CENPF in not merely tumor tissues but also cell lines in the carcinomas of the cervix. In vitro and vivo experimental investigations have demonstrated that the suppression of CENPF can impede cellular multiplication, migration, and invasion while inducing ferroptosis. The ferroptosis induced by CENPF inhibition in cervical cancer cell lines is likely mediated through the Nrf2/HO-1 pathway. The data herein come up with the opinion that CENPF may have a crucial role in influencing anti-cervical cancer effects by inducing ferroptosis via the triggering of the Nrf2/HO-1 signaling pathway.

## Introduction

Till 2018, cervical cancer (CC) was reportedly the second major reason of cancer death among young and middle-aged women, with more than 300,000 deaths worldwide [1, 2]. Since the mid-1970s, the survival rate of CC patients has not risen significantly. Although the morbidity of cervical cancer has decreased in the last few years, there has been a growth in distant-stage disease and cervical adenocarcinoma that cannot be detected by cytology [1]. Furthermore, despite advances in probing early-stage cervical cancer, some patients still experience recurrence [3]. Utilizing the classification system established by the Federation International of Gynecology and Obstetrics (FIGO), cervical cancer patients with stage IB-IIA have reported relapse rates ranging from 11 to 22%, while patients at FIGO stage IIB to IV have reported rates ranging from 28 to 64% [4]. Unfortunately, patients who experience advanced, metastatic, or recurrent cervical cancer face a bleak outlook, with survival rates at one year falling between 10 and 15%. This outcome is primarily due to the limited options after chemoradiotherapy [5]. Consequently, it is essential to investigate the molecular pathways fueling cervical cancer advancement to identify potential treatment target points for patients who have metastatic or recurrent cervical cancer [2].

In a previous bioinformatics analysis, we identified centromere protein F (CENPF) as a biological marker and therapeutic objective spot of CC [6]. CENPF was initially discovered in the nucleus of human cervical cells, and mainly aggregates at kinetochores in the late G2 stage of mitosis [7]. Its involvement in mitotic control, microtubule dynamics, and transcriptional regulation has been established [8]. Depletion of CENPF leads to aberrant mitotic assembly and chromosomal behavior [9]. Furthermore, overexpression of CENPF has been observed in varieties of cancers, including hepatocellular, gastric, breast, and lung, indicating its oncogenic nature [10–13]. It promotes bone metastasis of breast carcinoma cells and is an up-and-coming therapy targeting [13]. In hepatocellular carcinoma, overexpression of CENPF is predictive of acute prognosis and promotes disease progression [14]. Thus, CENPF likely functions as an oncogene in multiple cancers. However, little is known regarding the expression levels and function of CENPF in cervical cancer, or its mechanism of action. Ferroptosis, a recently identified type of programmed cell death brought about by iron accumulation and lipid peroxidation [15], is shown to play a part in certain oncogenic mutations and cervical cancer cells susceptibility to ferroptosis. For instance, certain oncogenic mutations increase the sensitivity of cancer cells to ferroptosis. Furthermore, there is evidence that cervical cancer cells are susceptible to ferroptosis [16, 17].

This paper presents an analysis of CENPF expression and its role of in human cervical carcinoma tissues and cell lines, and investigates the potential involvement of cervical. Through our research, we have gained new insights into the molecular theory of cervical cancer and recognize propitious diagnostic markers and therapy targeting.

## Materials and Methods

### Cell Culture and Patient Samples

The Hepatobiliary Surgery Laboratory of Qingdao Municipal Hospital in Shandong, China maintains several human CC cell lines including HeLa, SiHa, CaSki, and HT-3, as same as the normal human cervical epithelial cell line HcerEpic. These cells were incubated with Dulbecco’s Modified Eagle Medium (DMEM; Meilunbio, China) byintroducing 10% fetal bovine serum (FBS; Meilunbio, China) and 1% antibiotics (Penicillin-Streptomycin Solution, Beyotime, China) at 37 °C with 5% CO2 in a moist atmosphere. Tumor Tissue specimens from 70 CC patients and 20 cases of normal cervical tissue were collected with Ethics Approval No. 049 from the Ethics Committee of Qingdao Municipal Hospital, and all patients have subscribed informed consent document.

### Lentiviruses Construction and Cell Transfection

GENECHEM (Shanghai, China) constructed mouse small hairpin RNA (shRNA) sequences and lentiviral vectors encoding green fluorescent protein (GFP) targeting CENPF. The shRNA sequence was 5ʹ-GCAACCATCTACTTGAAGA-3’. The cell lines were transfected with the sh-CENPF and negative control shRNA (sh-NC) lentiviral constructs. The stably transfected cells were selected using 1 mg/mL puromycin and expanded for subsequent experiments.

### Immunohistochemistry

The tissue samples were set rigidly in 4% paraformaldehyde, sectionalized by paraffin embedding, and then cut into parts. The paraffin-embedded sections underwent deparaffinization using xylene, rehydration in anhydrous ethanol, and antigen retrieval through heating in citrate buffer solution. Following the quenched using 3% hydrogen peroxide in methanol of the endogenous, 5% goat serum drip to the sections (MXB, FuZhou, China) at room temperature condition to prevent non-specific binding. Tumor sections were hatched with anti-CENPF antibody (1:100) at 4 °C overnight and thereafter with goat anti-mouse/rabbit IgG polymer (PV-6000, ZSGB-BIO, China). The staining was developed with DAB chromogenic solution and the sections were counterstained with hematoxylin. Using the AxioVision Rel.4.6 to take images by an computerized image-processing systems (Carl Zeiss AG., Jena, Germany).

### Western Blot

RIPA buffer solution (Beyotime, Shanghai, China) is used to lyse cell or tissues and added to ice with 1 mM PMSF (Beyotime, Shanghai, China) for 20 min. Proteins were isolated by SDS-PAGE (7.5–10%) and shifted to PVDF transfer membranes (Millipore, Bedford, MA, USA). After curding in 5% skim milk at ambient temperature for 2 h, then add primary antibodies against CENPF (1:200, TD2310M, Abmart Shanghai Co. Ltd., China), GPX4(1:2000, 67763-1lg, Proteintech Group Inc., Wuhan, China), XCT (1:2000, 26864-1-AP, Proteintech Group Inc., Wuhan, China), P53 (1:200, MH3771S, Abmart Shanghai Co. Ltd.,China), NRF2 (1:100, T551365, Abmart Shanghai Co. Ltd., China), HO-1(1:200, BOS1418BP328, Boster Biological Technology Co. Ltd., China), DMT1 (1:200, 20507-1-AP, Proteintech Group Inc., China), GAPDH (1:800, M20006S, Abmart Shanghai Co. Ltd., China) and β-tubulin (1:800, M30109S, Abmart Shanghai Co. Ltd., China) to them and hatched overnight at 4 degree Celsius. Next, the blots were cleaned through TBST solution and hatched with HRP-conjugated secondary antibodies (1:8000) at ambient temperature with 2 h. The bands were developed using ECL Plus chemiluminescent solution (Meilun, Dalian, China) and visualized by the Chemical Signal Observation Strip™ Gel Imaging Analysis System (Tanon4600SF, Shanghai, China). Image J software was used to quantify band intensities.

### Cell Viability and Proliferation Assay

Using the CCK-8 assay kit (K1018, APExBIO Technology LLC, TX, USA) to measure cell viability. In simple terms, the amount of 5000 cells per well suitably transfected cells were got into 96-well plates. At the appointed time, 10 µl CCK-8 solution was incorporation into each well and the cells were hatched for 2 h. The optical density of the wells at 450 nanometer wavelength was measured with Elx800 (BioTek, Winooski, Vermont, USA). To evaluate proliferation capacity, the cells were sowed into 6-well pates and cultivated for 10 days. The substrate was refreshed every two to three days and colonies that formed subsequently were immobilized with 4% paraformaldehyde, dyed with 0.1% crystal violet, and underwent manual counting.

### Wound Healing Assay

CC cells were cultivated in 6-well plates till they were 90% confluent. Using the tip of a sterile pipette to scratch the monolayer and then cleaned with PBS to remove the shed cells, and incubated for 24 h. The wound coverage area was imaged at 0 h and 24 h by using microscope, and cell relative mobility was calculated and finally computed by using the under formula: (W2-W1)/W1 × 100%.

### Cell Invasion Assay

In vitro cell invasive ability was evaluated by Transwell insert (Corning, USA). The chamber membranes were pre-coated with Matrigel (Corning Incorporated, USA). The CC cells were engrafted into the top chambers of the inlets in medium devoid of serum, while lower chambers involved 800 µl of complete substrate with 10% FBS as the chemotactic agent. After one day’s culture, number of transmembrane migrating or invading cells were dyed with 0.5% crystal violet and figured out.

### Measurement of Reactive Oxygen Species

Using the DCF-DA reagent kit (Cat # S0033S, Beyotime, Shanghai, China) to measure intracellular ROS levels. Tentatively, the cells were grafted into 6-well plates and cultivated for one day. Cells incubated with DCF-DA for 1 h were cleaned with PBS and collected using trypsin. The single-cell suspension was measure by the adoption of flow cytometry technology. The results were processed and analyzed with the FlowJo software.

### Malondialdehyde Assay

Using the MDA assay kit (Cat: S0131S, Beyotime, Shanghai, China) to detect the malondialdehyde levels of the cultured cells according to the instructions provided by the manufacturer. The degree of lipid peroxidation was measured by counting the amount of MDA relative to the total amount of protein.

### Transmission Electron Microscope

The cells were immobilized with 2.5% glutaraldehyde solution (pH 7.4) for 2 h, after that, three rinses with 0.1 M phosphate buffer solution (pH 7.2). Following that, they were immobilized with 1% osmotic acid solution for 2 h at 4 °C. Following that, the specimens underwent dehydration using a progressive series of ethanol and were subsequently encased in Epon-Araldite resin within molds. Ultra-thin sections were dyed with 3% uranyl acetate and 2.7% lead citrate after cutting from the blocks and were then observed and collected by HT7800 transmission electron microscopy.

### In Vivo Xenograft Model

Feeding food and water ad libitum to 5-week-old female BALB/ C-nu mice which were purchase from Beijing Vital River Laboratory Animal Science Co., LTD. The animals were placed at 25 degree centigrade under 12 h of alternating light and darkness. Each mouse was injected with 5 × 10^6^ SiHa cells transfected with sh-CENPF (KD) or sh-NC (control) into their backs. Seven days after inoculation of the suitable cells, the CENPF-KD group was injected intra-abdominally with the Nrf2 activator tertiary butylhydroquinone (TBHQ:10 mg/kg, MCE, China) or normal saline (*n* = 5 each). Saline was administered to mice in the control group (*n* = 5). The drug/saline was administered every other day for 21 days. Furthermore, the mice were monitored with regular weight and measurement assessments every 3 days. After 21 days of culture, the mice were executed and dissected to facilitate the removal of their tumor tissues for immunohistochemical and western blot.

### Statistical Analysis

Using GraphPad 7 (GraphPad Software Inc., CA, USA) to analyze all statistical data. Datas are expressed as mean ± standard deviation (SD) derived from a minimum of three independent trials. A two-tailed Student’s *t*-test (two-tailed) was employed to cotrast the data from the two sets, and a *p*-value < 0.05. The statistical significance was observed.

## Results

### *CENPF* is Hyper-expressed in Human Cervical Carcinoma Tissues and Cell Lines

**Fig. 1:**
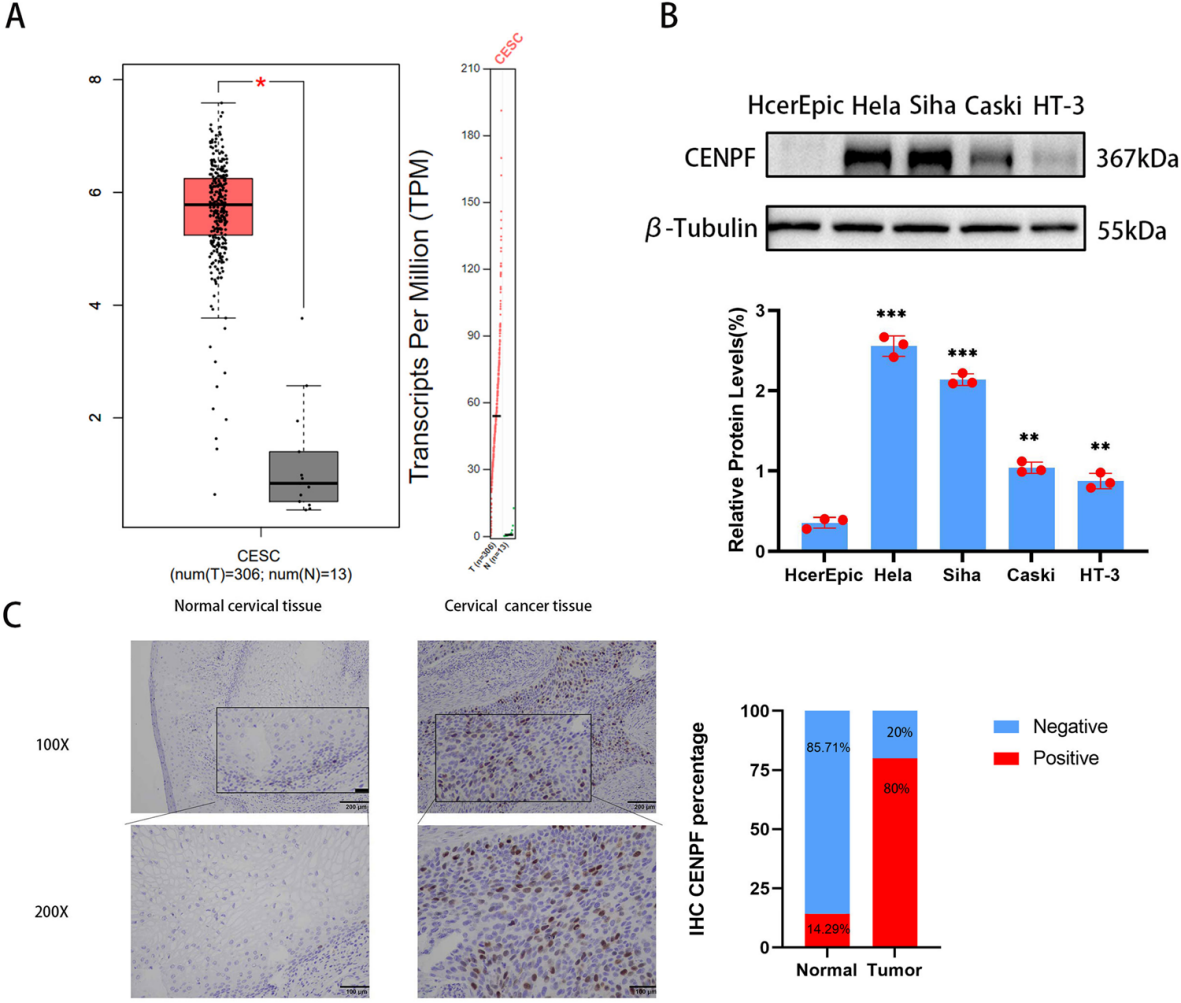
CENPF is significantly up-regulated in cervical cancer. **A** CENPF levels in cervical squamous cell carcinoma was analysed via GEPIA database. **B** Western blot showing CENPF protein level in normal cervical cell line (HcerEpic) and CC cell lines (HeLa, SiHa, CaSki, HT-3). **P < 0.01, ***P < 0.001 compared with HcerEpic. **C** Representative images of CC tissues and normal cervical tissue immuno-stained for CENPF (scale bar = 200 μm)

In the first place, an analysis of the GEPIA database revealed an elevated expression of CENPF in human cervical cancer tissues and cell lines. Specifically, Fig. 1A demonstrates the upregulation of CENPF in cervical squamous cell carcinoma relative to regular cervix. The CC cell lines (CaSki, HT-3, HeLa, and SiHa) also expressed significantly higher levels of the CENPF protein compared to a non-malignant human cervical cell line (HcerEpic) (Fig. 1B). Furthermore, the CC cell lines HeLa and SiHa expressed higher levels of CENPF compared to the cervical epithelial cancer cell lines CaSki and HT-3. Thus, the HeLa and SiHa cell lines were used for subsequent experiments. In addition, the expression of CENPF in the CESC and normal cervical tissues was analyzed by immunostaining. As seen in Fig. 1C, CENPF was significantly expressed in the CC tissues but barely detected in the ordinary cervix. These results indicated that CENPF possibly plays the carcinogenic role in cervical carcinoma.

### *CENPF* Knockdown Inhibits the Biological Functions of Cervical Cancer Cell

**Fig. 2:**
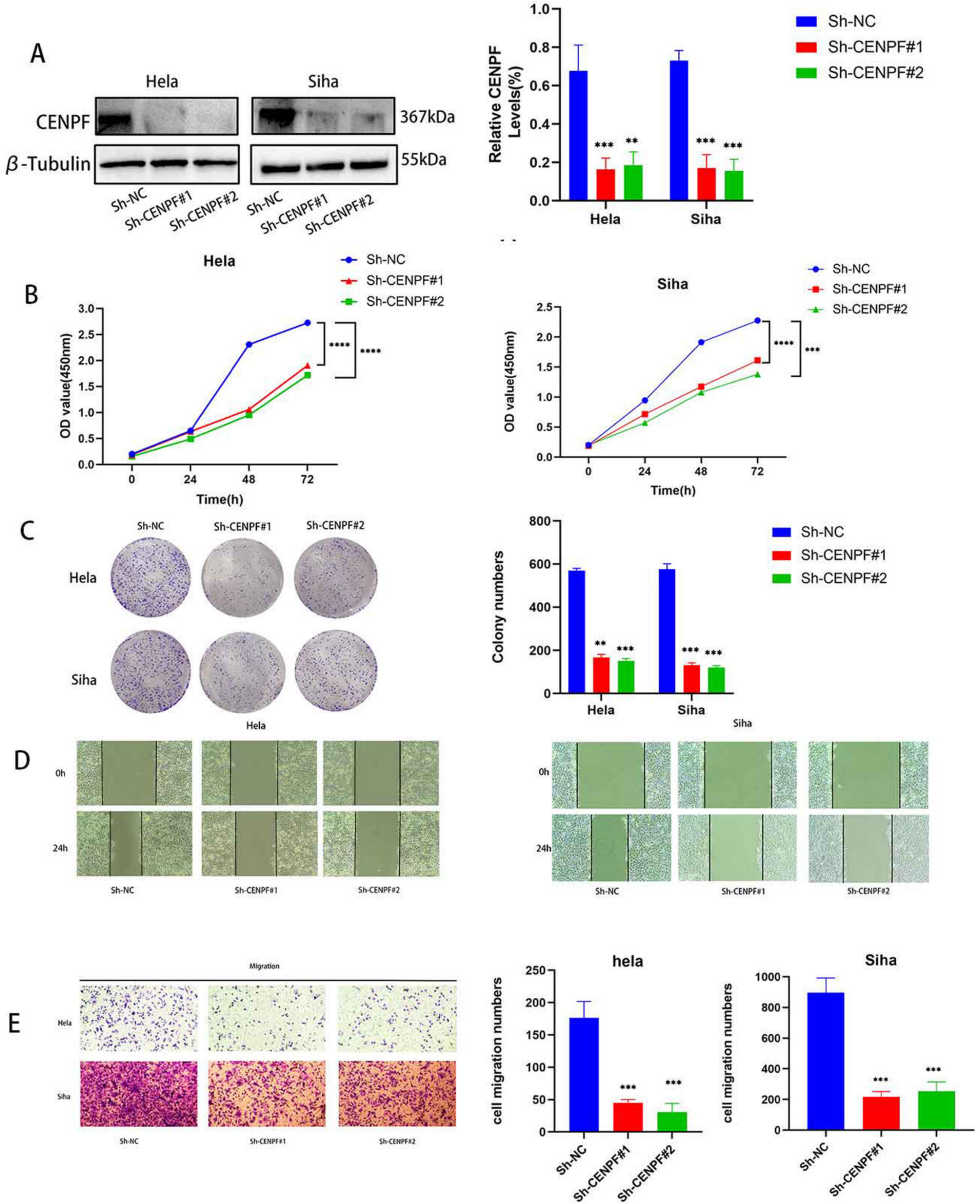
CENPF knockdown inhibited the malignant potential of CC cells and induced ferroptosis in vitro. **A** Western blot showing CENPF expression in HeLa and SiHa cells transfected with sh-NC and sh-CENPF. **B** Viability rates and **C** Number of colonies formed by the HeLa and SiHa cells transfected with sh-NC and sh-CENPF. **D** Migration rates of the HeLa and SiHa cells in the wound healing assays. **E** Invasion rates of the HeLa and SiHa cells in transwell assays respectively. Data are the mean ± SEM of at least three independent experiments. **P < 0.01, ***P < 0.001, ****P < 0.0001

For further research into the character of CENPF in cervical cancer, we used shRNA to construct knockout genes in HeLa cells and SiHa cells and identified that CENPF protein levels were decreased in both cell lines compared to the Sh-NC group (Fig. 2A). CENPF knockdown significantly decreased the viability of the CC cell lines and restrained their growth (Fig. 2B). Furthermore, the colony capacity of the HeLa and SiHa cells was reduced following CENPF knockdown (Fig. 2C). Wound healing experiments further showed that downregulated CENPF in HeLa and SiHa cells markedly inhibited their migratory ability in terms of the wound coverage rate (Fig. 2D). In the Transwell assay, HeLa and SiHa with CENPF knockdown showed reduced migration and invasion compared to the respective controls (Fig. 2E). Taken together, CENPF knockdown inhibited the malignant potential of CC cells.

### *CENPF* Knockdown Produced Ferroptosis in CC Cells Via Inhibition of the Nrf2/HO-1 Signaling Axis

**Fig. 3:**
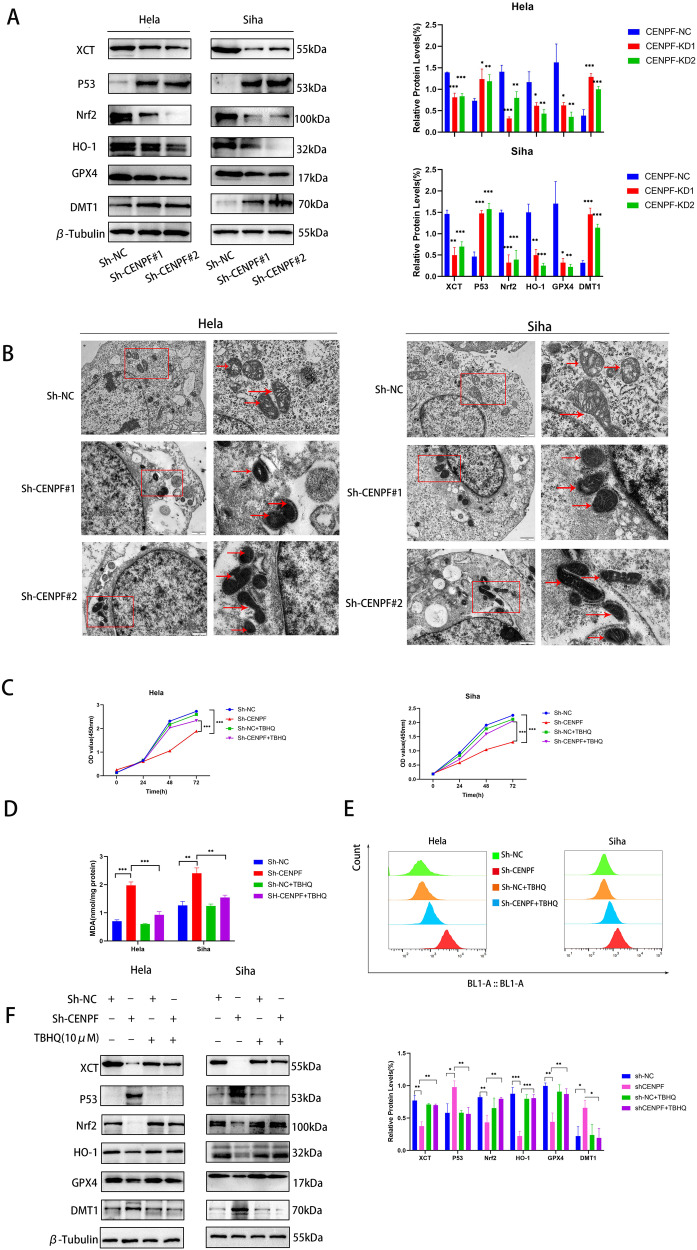
**A** Western blot showing GPX4, XCT, Nrf2, HO-1, p53 and DMT1 protein levels in HeLa and SiHa cells transfected with sh-NC and sh-CENPF. **B** Transmission electron microscopy images of HeLa and SiHa cells (scale bar = 500 nm). **C**-**F**. HeLa and SiHa cells were treated as in **B**. **C** Viability rates of CENPF-knockdown HeLa and SiHa cells with or without TBHQ treatment. *P < 0.05, **P < 0.01, ***P < 0.001, ****P < 0.0001. **D** The MDA levels relative to total protein content in the indicated groups. **E** ROS levels in the indicated groups. **F**. Western blot showing GPX4, XCT, Nrf2, HO-1, p53 and DMT1 protein levels in HeLa and SiHa cells. Data are the mean ± SEM of at least three independent experiments. *P < 0.05, **P < 0.01, ***P < 0.001

To delve deeper into the character of CENPF in CC cell ferroptosis, we analyzed several ferroptotic proteins’ expression level in the CENPF-knockdown and control cells. As depicted in Fig. 3A, glutathione peroxidase 4 (GPX4), solute carrier family 7 member 11 (xCT/SLC7A11), Nrf2 and HO-1 were downregulated, and p53 and metal transporter 1 (DMT1) were upregulated after CENPF knockdown. As shown in Fig. 3B, CC cells which were lacked CENPF had smaller mitochondria and higher mid-membrane density, which eventually led to the loss of their structural integrity and the development of mitochondrial matrix edema. Lipid peroxidation is an indicator of ferroptosis. Ferroptosis was evaluated by transmission electron microscopy. Consistent with this, we detected a significant growth in ROS and malondialdehyde (MDA) production in HeLa and SiHa cells with CENPF-knockdown (Fig. 3D, E). Investigations have shown that Nrf2 signaling axis has been recognized as a pivotal player in cancer progression and the inhibition of ferroptosis. To determine whether the induction of ferroptosis following CENPF knockdown was due to the inhibition of Nrf2/HO-1 signaling, we treated the CC cells with the NRF2 activator tert-butylhydroquinone (TBHQ). As shown in Fig. 3C, TBHQ (10 μM) improved the viability of the CENPF-knockdown HeLa and SiHa cells. Furthermore, TBHQ significantly suppressed ROS production and lipid peroxidation in the CENPF-knockdown cells compared to the untreated controls (Fig. 3D, E). The key ferroptotic proteins GPX4, XCT, Nrf2, and HO-1 were upregulated, whereas DMT1 and p53 were downregulated after TBHQ treatment. These findings indicate that CENPF protects CC cells against ferroptosis through downstream Nrf2 signaling, and the increased ferroptosis rates following CENPF knockdown are likely due to the inhibition of the Nrf2/HO-1 signaling axis (Fig. 3F).

### *CENPF* Knockdown Suppressed the Malignant Potential of CC Cells In Vivo

**Fig. 4:**
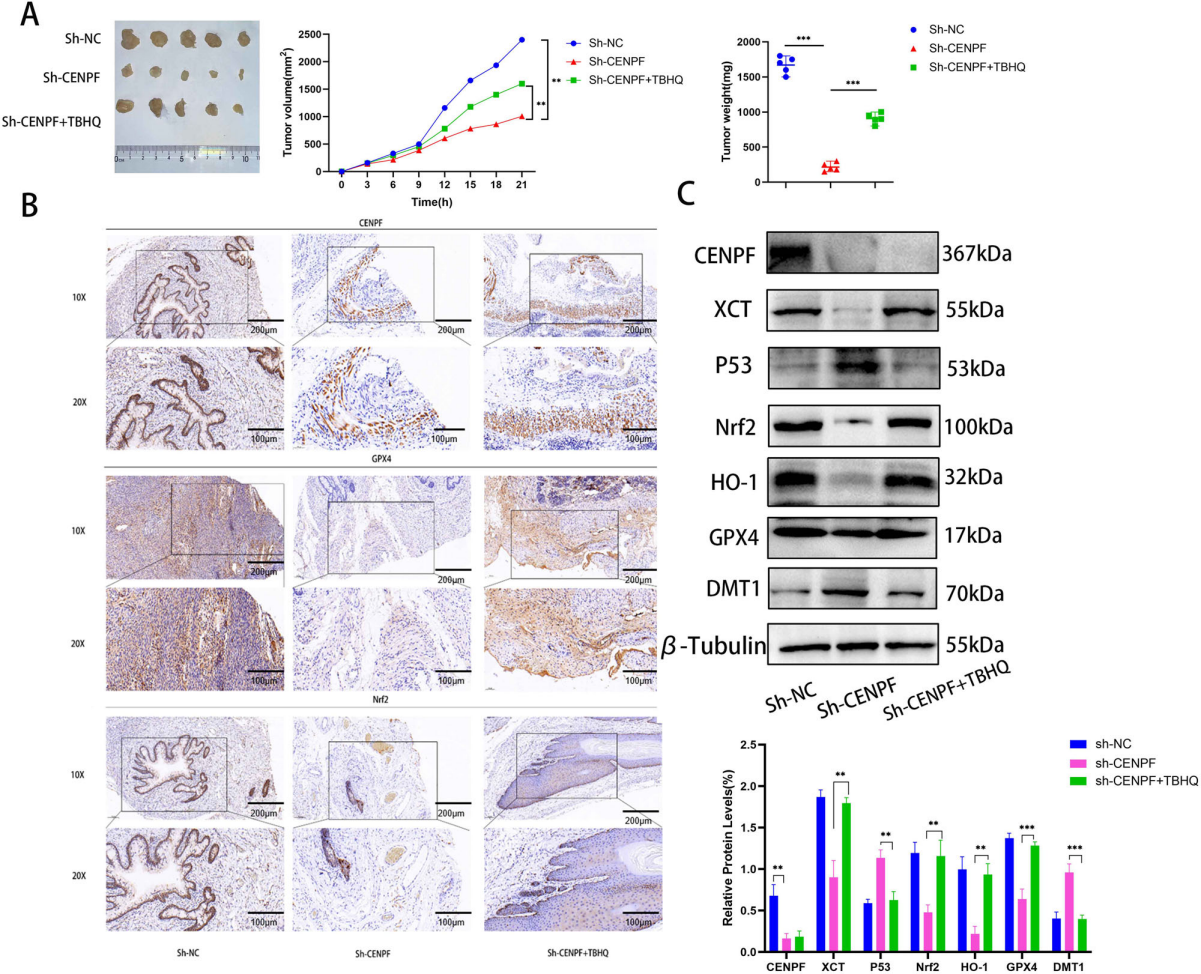
CENPF knockdown suppressed tumor growth in vivo. **A** Tumor volume and weight in the sh-NC, sh-CENPF and sh-CENPF + TBHQ gr.oups. **B** In situ expression of CENPF, GPX4 and Nrf2 in the subcutaneous tumor tissue sections form the indicated groups. **C** Western blot showing CENPF, GPX4, XCT, Nrf2, HO-1, p53 and DMT1 levels in the tumor tissues from the indicated groups. Data are the mean ± SEM of at least three independent experiments. **P < 0.01, ***P < 0.001

To interpret the in vitro findings, we conducted subcutaneous xenografts in nude mice using by control or CENPF-knockdown SiHa cells. Additionally, mice carrying sh-CENPF tumors were administered either saline or TBHQ (10 mg/kg body mass) to investigate the involvement of Nrf2 signaling in ferroptosis. Figure 4A displays that in comparison with the control group, sh-CENPF cells showed markedly smaller tumor sizes, and TBHQ treatment promoted the growth of Sh-CENPF tumors. Additionally, the sh-CENPF team exhibited noticeable reductions in the expression levels of CENPF, GPX4, and Nrf2 in neoplastic tissues when compared to the sh-NC team (Fig. 4B), and the expression levels of GPX4 and Nrf2 were restored by TBHQ (Fig. 4B). As shown in the Fig. 4C, GPX4, XCT, Nrf2, and HO-1 were upregulated, and DMT1 and p53 were downregulated in the TBHQ-treated mice.

## Discussion

Cervical cancer ranks as the fourth most prevalent cancer among women globally, resulting in over 300,000 deaths worldwide. Even though cervical cancer is preventable, it is recalcitrant to treatment due to the high rates of metastasis and recurrence. Therefore, a deeper exploration of the mechanisms of cervical cancer can pave the way for more valid targeted therapies [2]. In a preliminary bioinformatics study, we compared the gene chip and RNA-seq transcriptomic data of uterine cervix carcinoma and regular cervix and analyzed the correlation between differentially expressed genes and molecular characteristics. CENPF exhibited high expression levels in the cancer tissues and related to metastasis, the frequency of somatic mutations in tumor suppressor genes (TP53, MSH2, RB1), as well as genes implicated in tumorigenesis pathways, cell cycle regulation, and DNA damage and repair mechanisms [6].

The oncogene CENPF is a centromeric protein, and given its mitogen-specific expression and subcellular localization pattern, is also a potential marker of proliferation [9]. Furthermore, Jingbo Sun et al. observed that hyper-expression of CENPF in breast cancer was linked to poor prognostic and bone metastases [18], and Hongjin Chen et al. proposed the involvement of the CENPF-pERK-NEK2 pathway in HCC development [19]. However, the expression patterns, molecular mechanisms, and biological functions of CENPF in cervical cancer are not well explained. In this paper, we discovered that striking overexpression of CENPF in cervical cancer tissues compared to ordinary cervix. CENPF was first identified in the nucleus of HeLa cells and was associated with mitophagy [7]. Consistent with this, the CC cell lines HeLa and SiHa exhibited higher expression levels of CENPF compared to normal cervical epithelial cell lines. Furthermore, knocking down CENPF in the CC cells could attenuate the proliferation, migration, and invasion of CC.

There is demonstration that cervical cancer cells are extremely susceptible to ferroptosis, and activation of ferroptotic pathways can be a viable strategy to target these cells [20]. The following studies provide a brief summary of how hypoxia regulates HIF1α transcription [21], propofol enhances paclitaxel-induced cervical cancer cell ferroptosis [22]. Bioinformatics studies examining ferroptosis-related genes in malignancies have reported found that ferroptosis is closely linked to the cell cycle [23, 24]. Based on these findings, we hypothesized that the cell death induced by CENPF knockdown in our study is mediated via ferroptosis. Transmission electron microscopy revealed that cells with CENPF deficiency had smaller mitochondria and higher mid-membrane density. The loss of structural integrity and mitochondrial matrix edema were indicative of ferroptosis. Several genes including GPX4, SLC7A11, P53, Nrf2, HO-1 [25], and DMT1 [26] were associated with ferroptosis. We found that CENPF knockdown in the HeLa and SiHa cells downregulated GPX4, SLC7A11, Nrf2, and HO-1, and upregulated SLC7A11, P53, and DMT1. Iron accumulation and lipid peroxidation are the two main biochemical events of ferroptosis that lead to extreme ROS manufacture and pursuant cell death [27]. We detected high levels of ROS and the lipid peroxidation product MDA in the HeLa and SiHa cells with CNEPF knockdown. Nrf2 is a crucial transcription element that adjusts the antioxidant stress response, and can also protect cancer cells from ferroptosis [28, 29]. TBHQ, an activator of Nrf2, can effectively inhibit ROS production [30–32]. In our research, we have noted a noteworthy suppressive impact of TBHQ on both ROS generation and lipid peroxidation in CC cells after CENPF knockdown. TBHQ could effectively elevate the expression of XCT, GPX4, and other related markers p53, and Nrf2, HO-1, as compared to the control team. These findings indicated that Nrf2 lies downstream of CENPF, and the Nrf2/CENPF axis protects cervical cancer cells against ferroptosis.

We also established a subcutaneous xenograft model using SiHa cells and found that CENPF knockdown inhibited tumor growth compared to the sh-CENPF group, whereas TBHQ treatment accelerated the tumor growth rate. Furthermore, CENPF, GPX4, XCT and Nrf2 proteins were meaningfully down-expressed in the Sh-CENPF tumors in comparison to the Sh-NC group. However, TBHQ improved the expression of GPX4, XCT, Nrf2, and HO-1, and reduced that of DMT1 and p53 in the CENPF-knockdown tumors. Taken together, the knocking down of CENPF restrained the malignant potential of cervical carcinoma cells by restraining the Nrf2 signaling axis.

## Conclusion

**Fig. 5:**
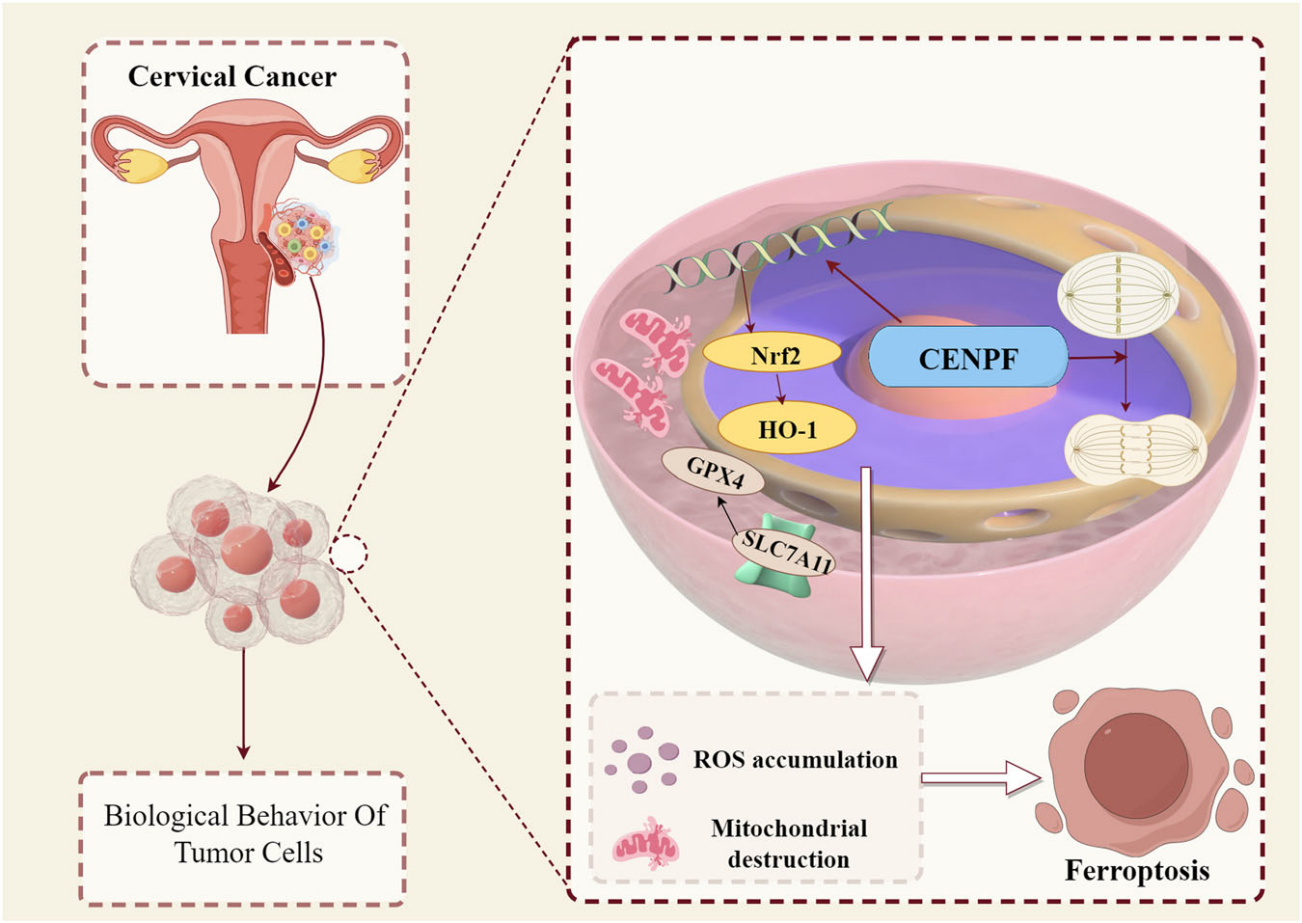
Schematic representation of the biological mechanism of action of CENPF on cervical cancer cells.

The conclusions of this research are summarized in Fig. 5. Our research group firstly confirmed that the CENPF is overexpressed in cervical neoplastic tissues. It is critical for the proliferation and migration of cervical cancer cells. Knocking down CENPF caused ferroptosis to happen in the cervical cancer cells by inhibiting the Nrf2/HO-1 signaling axis. Thus, CENPF is a hopeful therapeutic molecular target for cervical cancer that it is necessary to conduct further research.

## Data Availability

No datasets were generated or analysed during the current study.
